# Processability and Degradability of PHA-Based Composites in Terrestrial Environments

**DOI:** 10.3390/ijms20020284

**Published:** 2019-01-12

**Authors:** Patrizia Cinelli, Maurizia Seggiani, Norma Mallegni, Vito Gigante, Andrea Lazzeri

**Affiliations:** Department of Civil and Industrial Engineering, University of Pisa, Largo Lucio Lazzarino 2, 56122 Pisa, Italy; norma.mallegni@gmail.com (N.M.); vito.gigante@dici.unipi.it (V.G.); andrea.lazzeri@unipi.it (A.L.)

**Keywords:** biocomposites, natural fibers, poly(3-hydroxybutyrate-3-hydroxyvalerate), biodegradation, impact properties

## Abstract

In this work, composites based on poly(3-hydroxybutyrate-3-hydroxyvalerate) (PHB-HV) and waste wood sawdust (SD) fibers, a byproduct of the wood industry, were produced by melt extrusion and characterized in terms of processability, thermal stability, morphology, and mechanical properties in order to discriminate the formulations suitable for injection molding. Given their application in agriculture and/or plant nursery, the biodegradability of the optimized composites was investigated under controlled composting conditions in accordance with standard methods (ASTM D5338-98 and ISO 20200-2004). The optimized PHB-HV/SD composites were used for the production of pots by injection molding and their performance was qualitatively monitored in a plant nursery and underground for 14 months. This study presents a sustainable option of valuation of wood factory residues and lowering the production cost of PHB-HV-based compounds without affecting their mechanical properties, improving their impact resistance and biodegradability rates in terrestrial environments.

## 1. Introduction

Petroleum-based plastics are light, strong, durable, and demonstrate good resistance to degradation [1]. They offer a wide range of applications in the domestic, medical, and industrial fields in the form of single-use gears, packaging, furniture, machine chassis, and accessories to improve life quality [2]. For these reasons, approximately 150 million tons of plastic are consumed worldwide each year and this consumption is expected to continue growing until 2020 [3]. 

Large-scale dependence on petroleum-derived plastics leads to serious pollution problems; the methodologies used for plastic waste disposal are challenging. In landfills, the degradation rates are tremendously slow [4,5]. Incineration generates harmful by-products if advanced combustion designs, optimized operating practices, and effective emission-control technologies are not adopted. Chemical and mechanical recycling is not always possible, requires an advanced collection system, and causes a deterioration of the properties of plastic materials compromising their reuse [6,7]. 

Given this scenario, with the aim of decreasing plastic environmental impact, the use of bio-based and/or biodegradable polymers, such as polylactide acid (PLA), aliphatic polyesters, and polyhydroxyalkanoates (PHAs), having similar physicochemical properties as conventional plastics represent a valuable solution to the current plastic pollution problem [8,9,10,11]. However, the replacement of nondegradable with biodegradable plastics requires the complete knowledge of the biodegradability of these plastics and their blends in controlled and uncontrolled environments to have a real positive environmental impact. It is necessary to know their biodegradability and biodegradation rates both in managed (industrial and home composting, anaerobic digestion) and unmanaged (soil, oceans and rivers) environments for postconsumer management. Only a few biopolymers (PHAs and thermoplastic starch, TPS) are degradable in a wide range of managed and unmanaged environmental conditions; whereas most of them (e.g., PLA, polycaprolactone (PCL), poly(butylene succinate) (PBS), and polyhydroxyoctanoate (PHO)) degrade only in a narrower set of environmental conditions [12]. Narancic et al. [12] identified synergies but also antagonisms between polymers in blends that affect their biodegradability and biodegradation rates in different environments. Consequently, biodegradable plastics such as PHAs that degrade in a wide variety of controlled and uncontrolled environments are the most promising candidates for terrestrial and marine applications where their release into the environment does not cause plastic pollution. PHAs are a family of polyesters of several *R*-hydroxyalkanoic acids, synthetized by several microorganisms in the presence of excess carbon and when essential nutrients such as oxygen, nitrogen, or phosphorus are limiting or after pH shift [13,14,15,16,17,18]. PHAs have thermoplastic properties similar to those of polypropylene, good mechanical properties, and excellent biodegradability in various ecosystems [19,20,21,22,23,24,25]. The most common PHAs are the poly([R]-3-hydroxybutyrate) (PHB) and its copolyester with [R]-3-hydroxyvalerate (PHB-HV), which is well suited for food packaging [21]. Despite their good properties and excellent biodegradability, their relatively high cost (€7–12/kg) [14], compared to other biopolymers such as poly-lactic acid (PLA) (€2.5–3/kg) [26], has limited their use in commodities such as packaging and service items, restricting their use to high-value applications in the medical and pharmaceutical sectors. Efforts have been made to incorporate low-value materials, such as starch, into PHAs in order to reduce the cost of the final products [27,28,29]. Waste lignocellulose fibers, highly-available and at low-cost, sourced from agricultural and industrial crops, have been investigated as fillers in PHA-based composites [30,31,32,33,34].

In some previous cases, the developed composites displayed promising mechanical and physical properties. Natural fiber-reinforced composites show higher degradation when subjected to outdoor applications compared with composites with synthetic fibers [35]. Biodegradation of a composite occurs with the degradation of its individual components as well as with the loss of interfacial strength between them [36]. The weak interfacial bonding between highly polar natural fiber and non-polar matrix can lead to a reduction in final properties of the composite, ultimately hindering their industrial usage. Different methods have been applied to improve the compatibility and interfacial bond strength, including the use of various surface modification techniques [37,38]. It is harder to obtain high degrees of alignment with natural fibers than for synthetic fibers, since during the extrusion process, the long natural fibers tend to twist randomly [39]. This behavior compromises the mechanical properties of the composite as most of the natural fibers are not aligned parallel to the direction of the applied load. 

Composites containing sawdust fibers and petroleum-based plastics have been well-studied. For example, they were processed by Sombatsompop et al. [40,41] with polypropylene (PP) and polyvinylchloride (PVC), revealing a reduction in the overall strength and toughness of the composites with increasing the wood fiber content. In a previous work [42], composites based on PHB-HV and fibers of *Posidonia oceanica*, a dominant Mediterranean seagrass, were successfully produced by melt extrusion and their degradability was investigated in sea water. The results showed an increase in the impact resistance of the composites with increasing fiber content. The presence of fibers favored the physical disintegration of the composite, increasing its biodegradation rate under simulated and real marine conditions.

In the present work, composites based on PHB-HV and waste sawdust fibers (SD), derived from the wood industry, were produced by extrusion and characterized in terms of processability, rheological, and mechanical properties. Given their use in terrestrial applications, biodegradation tests were completed on the developed composites under simulated composting conditions in accordance with standard methods and we preliminarily evaluated the degradability in soil of PHB-HV-SD-based molded specimens (pots with thickness of 1 mm). 

## 2. Results and Discussion 

### 2.1. Composite Processing

**Figure 1:**
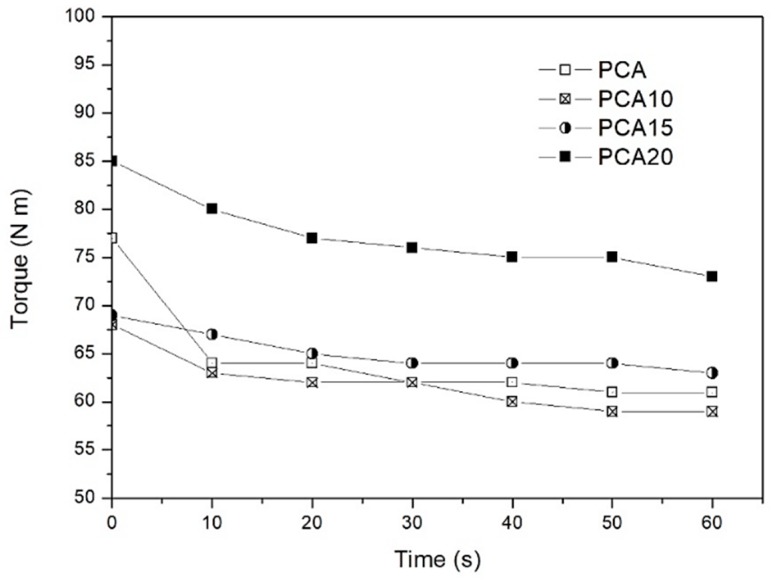
Torque-time curves obtained at 170 °C.

The torque measurements of polymeric melts characterize their flow behavior and reflect the trend of viscosity, as demonstrated by Melik et al. [43]. The objective of these measurements was to quantify the processing behavior of the investigated composites and, in particular, to evaluate the effect of the fibers on the melt fluidity. The average torque-time curves obtained at 170 °C and a rotor speed of 100 rpm with a HAAKE MiniLab are reported in Figure 1 for the PCA and PHB-HV/SD composites.

As shown, the incorporation of the SD fibers in the PHB-HV-based compound increased the torque and, consequently, the energy required for the melt mixing. With up to 15 wt % of fibers, there was a moderate increase in torque with respect to the pure matrix, whereas for PCA20, the torque values almost doubled, significantly affecting the processability of the biocomposite. 

### 2.2. Composite Characterization

**Figure 2:**
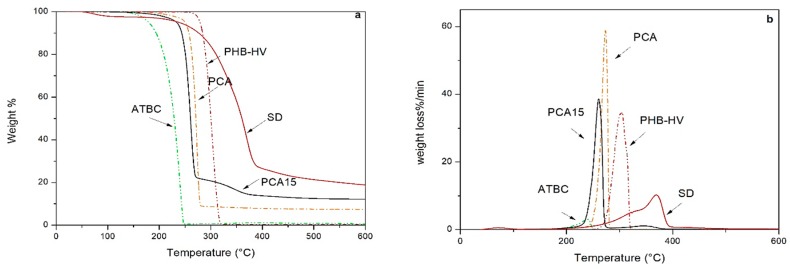
(**a**) TG and (**b**) DTG curves of the SD fibres, PHB-HV, ATBC, and developed composites.

The thermogravimetric (TG) and their derivative (DTG) curves of SD fibers, PHB-HV, ATBC, and the developed composites are shown in Figure 2.

SD fibers showed an initial weight loss at around 100 °C, attributable to loss in the residual humidity. Then, the sharp decrease observed at 250–350 °C can be related to the degradation of hemicellulose, cellulose, and lignin. Hemicellulose decomposes easily with respect to the other components. Typically, the pyrolysis of hemicellulose occurs between 200 and 280 °C, resulting in formation of CO, CO_2_, condensable vapors, and organic acids [44]. 

Therefore, the thermal degradation of the SD fibers occurs at temperatures higher than 200 °C, confirming their suitability of being processed with thermoplastic polymer matrices, such as PHB-HV, without thermal degradation occurring.

PHB-HV showed the onset of degradation at about 260 °C with maximum weight loss rate at 305 °C and no residue was recorded above 350 °C. For all of the produced composites, the thermal degradation started at temperatures above 200 °C, with the main degradation peaks occurring above 250 °C. 

The composite mechanical properties are shown in Table 1. 

The results of tensile tests showed that the elastic modulus was almost constant, except in the case of PCA20 where a higher fiber content led to a significant increase in stiffness, causing a decrease in break stress and elongation. This behavior is typical of composites with poor or no compatibility between the components, preventing the stress transfer phenomena from occurring and the filler particle becomes a stress concentrator, leading to brittle fracture [45].

**Figure 3:**
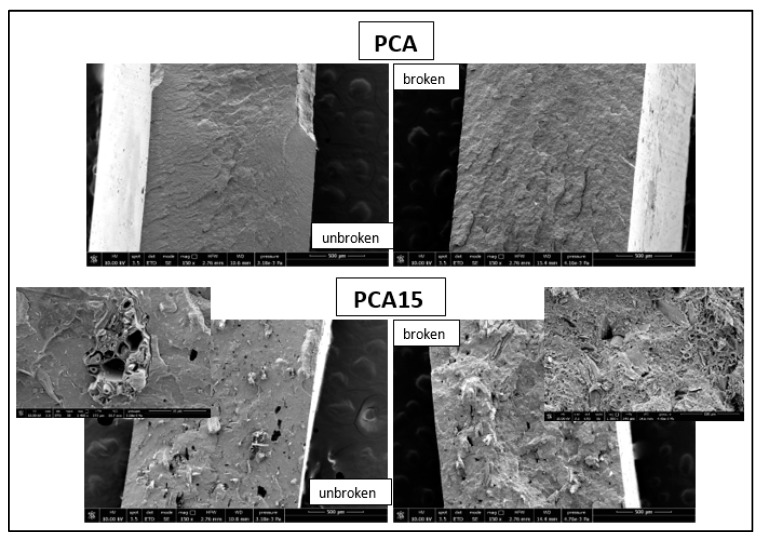
SEM images of the cross-sections of the PCA and PCA15 specimens before (unbroken samples) and after tensile tests (broken samples).

To better understand these results, SEM analysis was performed on the cross-section of the specimens before the tensile test and on the fractured sections obtained after testing (Figure 3).

The unbroken PCA15 specimen showed a good dispersion of SD fibers that are homogeneously distributed within the thermoplastic matrix. In the broken specimen section, it was possible to observe a significant fiber pullout. This means that the interfacial interactions between the fibers and the matrix were not sufficiently strong to maintain their cohesion during the tensile test.

Natural fibers are rich in cellulose, hemicellulose, lignin, and pectin; consequently, they tend to be active polar hydrophilic materials, whereas polymeric materials are nonpolar and show considerable hydrophobicity. The hydrophilic nature of natural fibers reduces the adhesion to a hydrophobic matrix and, as a result, a loss in strength may be induced. To prevent this, the fiber surface can be modified by several methods proposed in the literature, such as graft copolymerization of monomers onto the fiber surface, the use of maleic anhydride copolymers, alkyl succinic anhydride, stearic acid, etc. [38,46,47]. Therefore, a compatibilization method may be necessary to produce composites with tailored tensile properties.

Even without compatibilizers, interesting results were obtained from the impact test, in which the PCA15 compound showed a higher impact resistance compared with the matrix without fibers. Factors such as fiber/matrix de-bonding, fiber and/or matrix fracture, and fiber pull out improve the impact performance. Fiber fracture dissipates less energy compared to fiber pull-out, which is common in composites with strong interfacial bonds. A high impact energy is a sign of weak fiber/matrix bond [48,49]. In this case, the absorbed energy value increased up to 15 wt. % SD fibers, and then decreased at higher loadings, which caused an increase in material brittleness [50,51].

### 2.3. Biodegradability in Lab-Scale Terrestrial Environments

#### 2.3.1. Mineralization 

**Figure 4:**
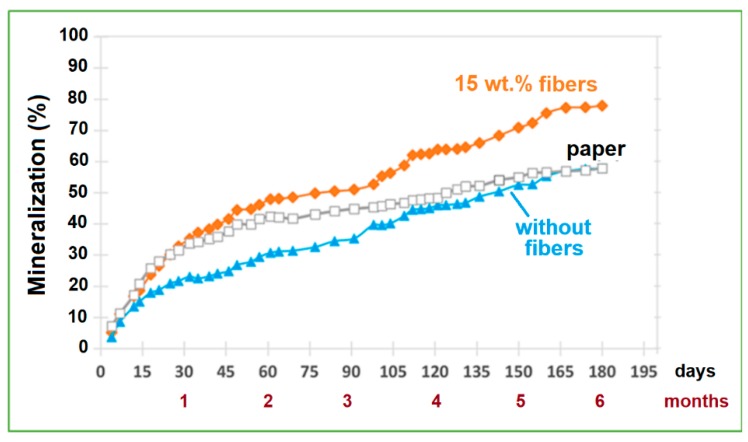
Mineralization curves under simulated terrestrial environmental conditions.

Figure 4 shows the aerobic biodegradation curves obtained on the lab-scale. As shown, after six months, the PCA15 composite reached a mineralization percentage of about 78%, higher than those of the control sample (filter paper) and the composite without fibers (58%). This behavior can be explained by the presence of the fibers favoring the disintegration of the sample, increasing its susceptibility to microbial attack. 

#### 2.3.2. Disintegration 

The average results of the disintegration test for each sample are reported in Table 2.

After 90 days, all the samples examined showed greater than 90% disintegration. The composite containing the higher amounts of fibers presented the highest degrees of disintegration, showing that the presence of fibers inside the matrix facilitated the degradation of the material according to the results of the mineralization test. 

Plasticizers, including ATBC, can accelerate the disintegration of polymeric matrices under composting conditions due to the greater mobility of the polymer chains, as observed by Arrieta et al. [52] for PHB-based blends. Narancic et al. [12] evidenced, in some cases, synergies between biodegradable polymers in blends that improve their biodegradation rates. A possible explanation of the synergy observed in some environments (e.g., home composting or anaerobic digestion) for PLA-PCL (80/20), PHB−PCL (60/40), and PCL−TPS (70/30) blends was that the addition of amorphous PCL, by lowering the crystallinity of the blends, improves their degradation rate.

Consequently, and also in this case, ATBC may have contributed, lowering the crystallinity of the PHB-HV together with the fibers to accelerate the disintegration of the specimens, thus increasing the biodegradation rate of the PHB-HV.

##### Degradation of Pots in Soil

**Figure 5:**
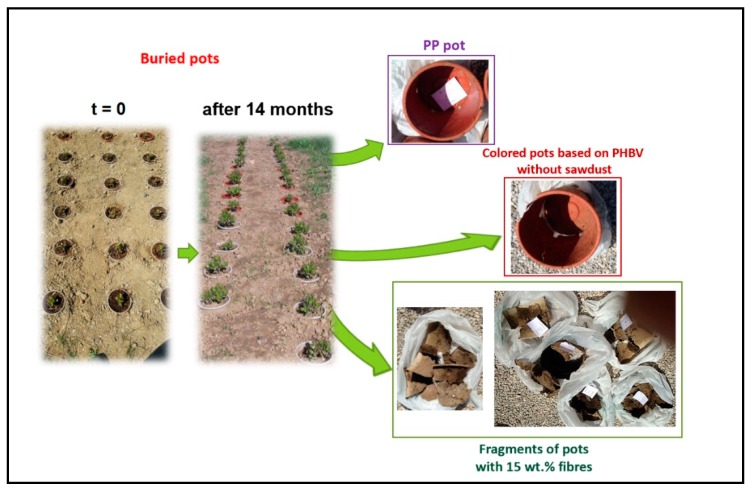
Buried PP and PHB-HV based pots without fibers and with 15 wt % sawdust fibers.

The performance and degradation of 180 pots (produced by injection molding with thickness of 1 mm and weight of about 115 g) was evaluated in three different environments: 60 were placed in a greenhouse (15 pots for each composite: PCA, PCA10, and PCA15, and 15 pots of PP, as reference); 60 pots were located outside on a cloth and not in contact with the soil (15 pots for each material); and 60 pots were buried into the soil up to the upper profile, leaving only the plants outside (Figure 5). 

After 14 months of experimentation, the PP pots showed no evident signs of degradation, as expected, whereas the pots based on PHB-HV showed initial signs of degradation, in particular those containing 15 wt % sawdust fibers. The plant growth performance was the same in all pots (PHB-HV, PHB-HV/SD, PP). 

Figure 5 shows the results obtained with the buried pots: the PP pots remained intact as expected. The pots based on PHB-HV without fibers were slightly damaged on the bottom, while the pots containing 15 wt % SD fibers were totally fragmented and markedly degraded, confirming the results of the lab-scale degradation tests. 

## 3. Material and Methods

### 3.1. Materials

PHB-HV (PHI002) with 5 wt % valerate content was supplied in pellets by Naturplast^®^ (Caen, France); it is characterized by a density of 1.25 g/cm^3^ and melt flow index (190 °C, 2.16 kg) of 15–20 g/10 min. According to the supplier data sheet, PHB-HV is a semi crystalline polymer with a glass transition temperature of around 5 °C and a melting temperature of around 155 °C. 

Sawdust fibers (SD) were obtained from a soft wood-processing company. SD was dried at 80° C in a vacuum oven for at least 24 h, then milled using a lab-scale grinder and screened with a 500-μm sieve. 

To improve the flexibility and processability of the composites with high content of fiber, a bio-based plasticizer, acetylbutylcitrate (ATBC), was used. ATBC was purchased from Tecnosintesi^®^ (Bergamo, Italy); it is a colorless liquid, soluble in organic solvents, produced by acetylation of tri-n-butylcitrate, having a density of 1.05 g/cm^3^, and a molecular weight of 402.5 g/mol. ATBC is widely used as food packaging as it is non-toxic. 

Micro-sized calcium carbonate particles, produced from a high purity white marble, with mean particle size (D_50_) of 2.6 μm and top cut diameter (D_98_) of 15 μm, cubic shape (aspect ratio = 1), and density of 2.7 g/cm^3^, provided by OMYA^®^ (Avenza Carrara, Italy) with trade name OMYACARB 2-AZ, were used as rigid inorganic filler. CaCO_3_ is one of the most commonly used fillers in thermoplastics to reduce both their production cost and to improve their properties, such as stiffness, impact strength, processability, and thermal and dimensional stability [53].

### 3.2. Composite Preparation

The composites (whose compositions by weight are reported in Table 3) were prepared by mixing the various components at 170 °C for one minute in a Thermo Scientific Haake microcompounder (HAAKE™, Karlsruhe, Germany) with a twin-conic screw system at 100 rpm speed. The labels PCA, PCA10, PCA15 and PCA20 indicate the composite without SD fibers (PHB-HV/CaCO_3_/ATBC 80/10/10 *w*/*w*/*w*%) and with 10, 15, and 20 wt % SD fibers, respectively. 

**Figure 6:**
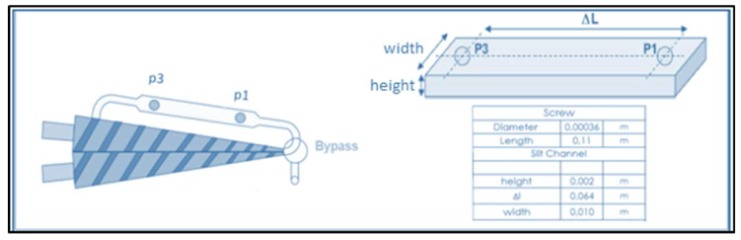
MiniLab Backflow channel and twin-screw conic system.

The miniLab compounder was equipped with a backflow channel (Figure 6) in which the recirculation of the melt occurs. During the melt mixing, the torque was recorded to evaluate the fiber effect on the melt fluidity. The extruded filaments were cut to obtain pellets used for the successive thermal characterizations, lab-scale biodegradation, and disintegration tests. 

For each formulation, tensile test specimens (Haake type 3 dog-bone bar) and Charpy Impact test samples (80 × 10 × 4 mm parallelepiped) were produced by feeding the molten material from the minilab into a mini-injection press (Thermo Scientific Haake MiniJet II, HAAKE™, Karlsruhe, Germany) at 210 bar with a mold heated at 60 °C.

Industrial compounding of the selected formulations was carried out using a COMAC EBC 50HT extruder (COMAC, Milan, Italy) with a temperature profile from 135 °C in the feeding zone to 170 °C at the screw terminal zone. The resultant pellets were then used to produce items such as pots using an injection molding machine (Negri Bossi VS250, Negri Bossi, Milan, Italy) at Femto Engineering (Florence, Italy). 

### 3.3. Composite Characterization

The thermal properties of the raw materials and composites in pellet form were evaluated by thermogravimetric analysis (TGA). These measurements were carried out, in duplicate, on 20 mg of sample using a Q500 TGA (TA Instruments; New Castle, DE, USA), under nitrogen flow (100 mL/min), at a heating speed of 10 °C/min from room temperature to 600 °C. 

Tensile tests were performed on the injection molded Haake Type 3 (557-2290) dog-bone tensile bars in accordance with ASTM D 638. Stress-strain tests were carried out at room temperature, at a crosshead speed of 10 mm/min, by an Instron 5500R universal testing machine (Canton, MA, USA) equipped with a 10-kN load cell, and interfaced with a computer running MERLIN Instron software, version 4.42 S/N–014733H (Instron^®^, Canton, MA, USA). 

The Charpy impact tests were completed at room temperature on 2-mm V-notched specimens using a 15 J Charpy pendulum (CEAST 9050, Instron^®^, Canton, MA, USA) following the standard method ISO 179:2000. For each mechanical test, at least five replicates were carried out. 

The morphology of the composite tensile specimens, before and after the tensile test, was investigated by scanning electron microscopy (SEM) using a FEI Quanta 450 FEG SEM (Thermo Fisher Scientific, Waltham, MA, USA). The SEM analysis was performed under high-vacuum conditions because air can inhibit the electron beam. The undamaged samples were frozen under liquid nitrogen and then fractured. The surfaces were metalized with gold using a Sputter Coater Edward S150B to avoid charge build up. 

### 3.4. Biodegradation in Terrestrial Environment

#### 3.4.1. Mineralization Test in Compost

**Figure 7:**
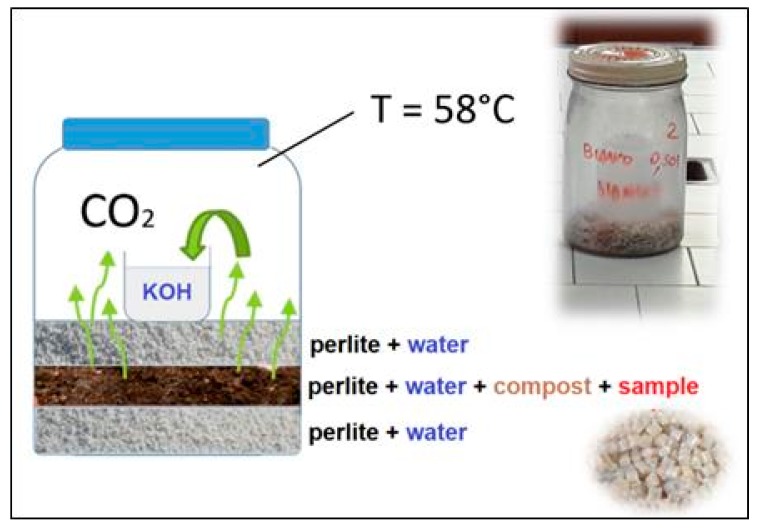
Scheme and photo of the apparatus used for the mineralization test.

Given the application of composites in terrestrial environments, a mineralization test in compost was carried out following a laboratory method [10] based on ASTM D5338 [54]. The setup of the system is shown in Figure 7. The test was carried out in glass vessels (1 L capacity) containing a layer of a mixture of 3 g compost, 4 g perlite, and 0.5 g of sample in form of pellets, wetted with 15 mL distilled water, in order to operate with a weight ratio of sample/compost of 1:6 in accordance with ASTM D5338. Finally, the mixtures were sandwiched between two layers consisting of 5 g perlite wetted with 20 mL of water. Perlite (tradename Agrilit 2), a hygroscopic aluminum silicate, was supplied in granules of 1–2 mm by Perlite Italiana Srl (Milan, Italy). Perlite is able to hold 3 to 4 times its weight in water, largely used in horticultural applications where it provides aeration and optimal moisture conditions for plant growth. In the mineralization test, the perlite mixed with the compost maintained the hydration of the medium and increased the contact surface between the sample and the inoculum of the compost. 

The used compost was derived from the organic fraction of solid municipal waste, kindly supplied by Cermec SpA, Consorzio Ecologia e Risorse (Massa Carrara, Italy). Compost had 23.8 wt % carbon content, 2.2 wt % nitrogen content, with a weight C/N ratio of 10.7. 

A 100 mL beaker was inserted in each test vessel containing 50 mL of 0.1 M KOH. The basic solution trapped the carbon dioxide evolved from the sample (represented schematically by the green arrows in Figure 7) on the basis of the following reaction (Equation (1)):

(1) $$2\mathrm{KOH}+{\mathrm{CO}}_{2}\to K_{2}{\mathrm{CO}}_{3}+H_{2}O$$

Every 2–5 days, the absorbing solution was titrated with 0.1 M HCl after addition of 0.5 N BaCl_2_ to avoid the presence of soluble carbonates. 

The amount of evolved carbon dioxide was evaluated using the following equation:

(2) $${\mathrm{mg}\;\mathrm{CO}}_{2}=\left(V_{\mathrm{KOH}}\cdot C_{\mathrm{KOH}}-V_{\mathrm{HCl}}\cdot C_{\mathrm{HCl}}\right)\cdot \frac{44}{2}$$

where mgCO_2_ is the CO_2_ evolved in mg from sample in a single test session; V_KOH_ is the volume in mL of KOH (50 mL) in flask at the beginning of test session; V_HCl_ is the volume in mL of HCl needed for titration of KOH at the end of test session; C_KOH_ and C_HCl_ are the concentrations of KOH and HCl in mol/L, respectively; 44 is molecular weight of CO_2_; and 1/2 is the stoichiometric coefficient (mol CO_2_/mol KOH) of the CO_2_ absorption reaction, as shown in Equation (1).

The mineralization extent of each sample was calculated as neat percentage (corrected for the inoculum endogenous emission from the blank vessels) of the overall theoretical CO_2_ (ThCO_2_) amount, calculated on the basis of the initial organic carbon content of the testing sample:

(3) $$\mathrm{Mineralization}\%=\frac{{\Sigma \mathrm{mgCO}}_{2}-{\Sigma \mathrm{mgCO}}_{2,\mathrm{blank}}}{{\mathrm{ThCO}}_{2}}\cdot 100$$

The mineralization test was carried out in triplicate and the average values are reported.

#### 3.4.2. Disintegration Test

According to ISO 20200 [55], the determination of the disintegration degree of polymeric material under simulated composting conditions [56] was carried out on PCA, PCA10, and PCA15 using a synthetic compost prepared by mixing the different components listed in Table 4. Then, tap water was added to the mixture to adjust its final water content to 50 wt % in total. 

**Figure 8:**
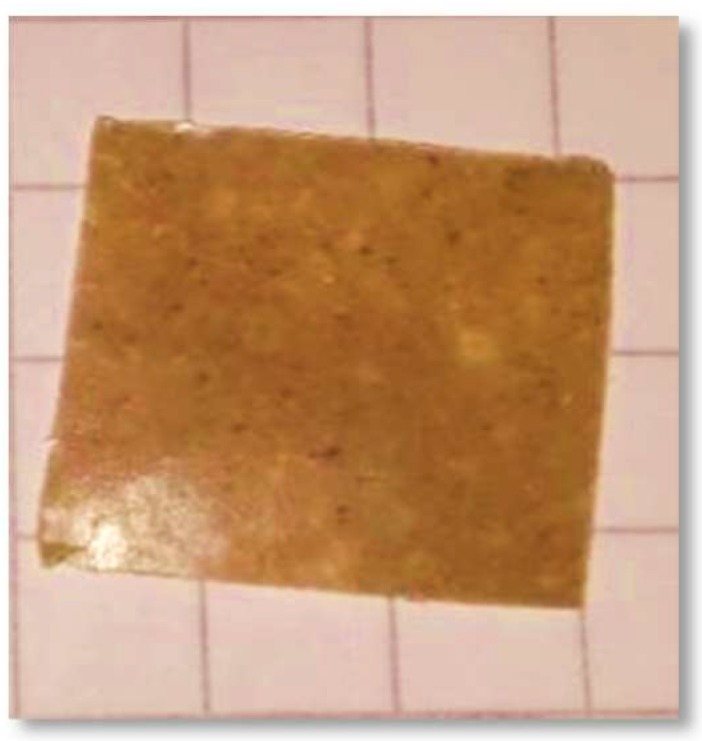
Sample used for the disintegration test.

Samples in the form of hot-pressed films (about 2.5 g) with the dimensions 2.5 × 2.5 cm and thickness of 1 mm (Figure 8) were used. Before mixing with the synthetic compost, the samples were dried in an oven at 40 ± 2 °C under vacuum for the time needed to reach constant mass. 

The test was carried out in flasks made of polypropylene, with dimensions of 30 × 20 × 10 cm (l, w, h), with two 5 mm holes (6.5 cm from the bottom) to provide gas exchange between the inner atmosphere and the outside environment. In each flask, 500 g of synthetic compost, mixed with three pieces of sample, were placed on the bottom, forming a homogeneous layer.

The flasks were covered with a lid ensuring a tight seal to avoid excessive evaporation and placed in an air-circulation oven at a constant temperature of 58 ± 2 °C for 90 days. Following the procedure reported in ISO 20200, the gross mass of the flasks filled with the mixture was determined at the beginning of the composting process and they were weighed at determined times and, if needed, the initial mass was restored totally or in part by adding distilled water. At the end of the test, the lid of each flask was removed and the flasks were placed in the air-circulation oven at 58 ± 2 °C for 48 h to dry the content. Then, the compost of each reactor was sieved using three standard sieves (ISO 3310 [57]): 10 mm, 5 mm, and finally a 2 mm sieve. Any residual pieces of sample that did not pass through the sieves were collected, cleaned to remove the compost that covered them, and dried in an oven at 40 ± 2 °C under vacuum to constant mass. The total material recovered from the sieving procedure was considered to be non-disintegrated material. So, the degree of disintegration was calculated, in percent, using the Equation (4):

(4) $$\mathrm{Disintegration}\;\left(\%\right)=\frac{m_{i,\mathrm{sample}}-m_{f,\mathrm{sample}}}{m_{i,\mathrm{sample}}}\cdot 100$$

where m_i,sample_ and m_f,sample_ represent the initial sample mass and the final dry mass of the sample recovered after sieving, respectively.

The disintegration test was carried out in triplicate on each sample.

## 4. Conclusions

In this work, we explored the use of waste wood fibers in combination with PHB-HV to manufacture biodegradable wood plastic composites in accordance with the circular economy principles.

We produced composites based on PHB-HV and different amounts (10, 15, and 20 wt %) of soft wood sawdust, a byproduct of the wood industry, by extrusion in the presence of appropriate amounts of ATBC as a plasticizer and calcium carbonate as an inorganic filler. 

Using appropriate amounts of ATBC, smooth processing was achieved for up to 20 wt % f fibers, despite the reduction in the melt fluidity observed with increasing fiber loading. The tensile modulus remained almost constant up to a 15 wt % fiber content, whereas the tensile strength and the elongation slightly decreased by increasing the fiber content up to 20 wt %. The impact resistance of the composites increased markedly with increasing SD amounts: the Charpy’s impact energy increased from 3.6 (without fiber) to 12.2 kJ/m^2^ for the composite with 15 wt % fiber. The results of the mineralization and disintegration tests in compost showed that the developed composites are compostable in accordance with EN 13427:2000, and the presence of fibers favored the physical disintegration of the composite, increasing the biodegradation rate of the polymeric matrix. After six months in compost, the composite with 15 wt % fiber showed a biodegradability of 78% compared with 58% for the composites without fibers and the paper (control sample). After 14 months, the pots based on PHB-HV/SD retained adequate mechanical performance and physical integrity inside the plant nursery and the plant growth performance was the same as that in traditional polypropylene pots, but the buried pots containing 15 wt % fibers were completely fragmentated compared with those without fibers, confirming the accelerating effect of the fibers on the degradation of the polymeric matrix in soil. 

In conclusion, the developed composites based on PHB-HV and waste wood sawdust are particularly suitable for production by the extrusion of relatively low-cost items such as pots, which are compostable and biodegradable in soil and usable in agriculture or plant nursery.

## Figures and Tables

**Table 1:** Mechanical properties of the composites with different SD fiber contents.

Sample	Young’s Modulus (GPa)	Stress at Break (MPa)	Elongation (%)	Charpy Impact Energy (kJ/m^2^)
PCA	2.64 ± 0.28	25.62 ± 2.11	2.14 ± 0.50	3.57 ± 0.36
PCA10	2.35 ± 0.24	21.02 ± 0.94	2.05 ± 0.28	6.17 ± 0.24
PCA15	2.52 ± 0.15	18.52 ± 0.84	1.35 ± 0.14	12.24 ± 0.50
PCA20	2.94 ± 0.35	20.93 ± 1.57	1.35 ± 0.13	5.91 ± 0.40

The values are the mean ± SD of at least five determinations.

**Table 2:** Average percentage of disintegration after 90 days under simulated composting conditions.

Sample	Sample No.	Disintegration (%)	Average Disintegration (%)
PCA	1	87.2	92.6
	2	94.3	
	3	96.4	
PCA10	1	83.4	93.2
	2	100.0	
	3	96.2	
PCA15	1	96.3	94.2
	*2*	88.6	
	*3*	97.7	

**Table 3:** Composite formulations.

	Weight Percentage			
Composite	PHB-HV	CaCO_3_	ATBC	Sawdust Fibers
PCA	80	10.0	10.0	0
PCA10	72	9.0	9.0	10
PCA15	68	8.5	8.5	15
PCA20	64	8.0	8.0	20

**Table 4:** Compost composition.

Material	Dry Mass %
Sawdust	40
Rabbit-feed	30
Ripe compost	10
Corn starch	10
Saccharose	5
Cornseed oil	4
Urea	1
Total	100
